# Study design: policy landscape analysis for sugar-sweetened beverage taxation in seven sub-Saharan African countries

**DOI:** 10.1080/16549716.2020.1856469

**Published:** 2021-01-21

**Authors:** Anne-Marie Thow, Agnes Erzse, Gershim Asiki, Charles Mulindabigwi Ruhara, Gemma Ahaibwe, Twalib Ngoma, Hans Justus Amukugo, Milka N. Wanjohi, Mulenga M. Mukanu, Lebogang Gaogane, Safura Abdool Karim, Karen Hofman

**Affiliations:** a Menzies Centre for Health Policy, School of Public Health, The University of Sydney, Sydney, Australia; b SAMRC Centre for Health Economics and Decision Science - Priority Cost Effective Lessons for Systems Strengthening (PRICELESS SA), University of the Witwatersrand, School of Public Health, Johannesburg, South Africa; c School of Economics, University of Rwanda, Butare, Rwanda; d Economic Policy Research Centre (EPRC), Makerere University, Kampala, Uganda; e Oncology Department, Muhimbili University of Health and Allied Sciences, Dar Es Salaam, Tanzania; f Community Health Department, School of Nursing, Faculty of Health Sciences, University of Namibia, Windhoek, Namibia; g Health and Systems for Health Unit, African Population and Health Research Center, Nairobi, Kenya; h Health Policy and Management Unit, University of Zambia School of Public Health, Lusaka, Zambia; i Department of Health Promotion & Education, Boitekanelo College, Gaborone, Botswana

**Keywords:** Noncommunicable disease, tax, sugar-sweetened beverage, political economy, policy

## Abstract

This paper reports on the design of a study to examine the policy landscape relevant to sugar-sweetened beverage taxation in seven sub-Saharan African countries. The study responds to the need for strong policy to address the rising burden of non-communicable diseases in the region. Sugar-sweetened beverage taxation has been widely recommended as a key component of a comprehensive policy approach to NCD prevention. However, it has proved a contentious policy intervention, with industry strongly opposing the introduction of such taxes.

The aim was to identify opportunities to strengthen sugar-sweetened beverage taxation-related policy for the prevention of nutrition-related NCDs in a subset of Eastern and Southern African countries: Kenya, Tanzania, Botswana, Rwanda, Namibia, Zambia, Uganda. The study was conducted as a collaboration by researchers from nine institutions; including the seven study countries, South Africa, and Australia. The research protocol was collaboratively developed, drawing on theories of the policy process to examine the existing availability of evidence, policy context, and stakeholder interests and influence.

This paper describes the development of a method for a policy landscape analysis to strengthen policies relevant to NCD prevention, and specifically sugar-sweetened beverage taxation. This takes the form of a prospective policy analysis, based on systematic documentary analysis supplemented by consultations with policy actors, that is feasible in low-resource settings. Data were collected from policy documents, government and industry reports, survey documentation, webpages, and academic literature. Consultations were conducted to verify the completeness of the policy-relevant data collection. We analysed the frames and beliefs regarding the policy ‘problems’, the existing policy context and understandings of sugar-sweetened beverage taxation as a potential policy intervention, and the political context across relevant sectors, including industry interests and influence in the policy process.

This study design will provide insights to inform public health action to support sugar-sweetened beverage taxation in the region.

## Background

The burden of non-communicable diseases (NCDs) across Africa has risen to be almost equivalent to the total burden associated with communicable, maternal, neonatal, and nutritional diseases [1]. Cardiovascular diseases were the main cause of death attributing up to 57% of deaths in the African Region [2]. NCDs are projected to be the leading cause of death by 2030 in the region [3]. With limited resources available for health-care systems, there is an urgent need to prioritise prevention of NCDs for public health [4]. In addition, the morbidity and mortality associated with NCDs among the working-age population means that NCDs present a challenge to economic growth and development in the region [1]. The burden of NCDs also falls most heavily on people of low socioeconomic status, and high health-care costs can further contribute to poverty and economic inequity [5].

The changing development and nutrition context has contributed to rising rates of NCDs in sub-Saharan Africa. Fruit and vegetable consumption is declining in many countries [6]. Consumption of processed foods and beverages, often high in salt, fat, and sugar, has risen substantially in most SSA countries, although it is still quite low relative to wealthier countries [7]. For example, in Uganda, processed foods account for 44% of the value of foods consumed, compared to 90% in South Africa, with the remaining predominantly unprocessed and home-grown food [8]. These dietary patterns are known independent risk factors for NCDs, and are associated with an increased risk of overweight and obesity (which in turn are risk factors for NCDs). [9,10].

Proactively addressing risk factors will play a crucial role in limiting the rise of NCDs in SSA. Taxes on sugar-sweetened beverages (SSBs) have been widely recommended as part of comprehensive policy approaches to prevent nutrition-related NCDs, and are an effective strategy to reduce consumption of SSBs [11,12]. SSB consumption is associated with an increase in metabolic risk factors for diabetes and cardiovascular disease, including hypertension, weight gain, high blood lipid, and blood glucose concentrations as well as non-alcoholic fatty liver disease [13–17]. Available data suggest that consumption of SSBs is rising in the region, and the SSB industry has identified sub-Saharan African countries as a growth market, due to rising incomes, rapid economic growth, and a large youthful population [18,19]. SSBs are heavily marketed, and increases in consumption have been particularly noted among children and adolescents [20].

Although there has been growing recognition of the importance of NCD prevention in SSA, the adoption and implementation of NCD prevention policy has faced challenges. Recent research on NCD policy development and intersectoral governance in Kenya, South Africa, Cameroon, Nigeria, and Malawi found low levels of public and decision-maker awareness of the problem of NCDs, weak political will, and inadequate resources [21]. Politico-economic factors also hamper the adoption of NCD prevention policies that affect industry actors, such as SSB taxes.

Beverages are one of the fastest-growing packaged foods categories in SSA [18]. Increasing vertical and horizontal integration of the beverage industry, including acquisition of regional companies by multi-national corporations, means the SSB industry is also growing in political influence due to an increased visibility of their contribution to economic growth [19,22]. For example, South Africa’s SSB tax was strongly opposed by industry actors, who actively opposed policy makers based on their contribution to economic growth and employment, and the potential negative impacts of a tax on the industry (37,38). The economic contribution of the SSB industry to other countries in the region, particularly in relation to employment, is rising, making this a potential point of tension for public health efforts to curb consumption [23]. Although emerging international evidence suggests that the economic impacts of SSB taxation are minimal, such arguments are politically powerful [24].

This paper presents the method for a policy landscape anlaysis: a prospective desk-based policy analysis of SSB taxation and policies related to the potential use of revenue to promote access to healthy foods, with an explicit focus on political economy factors. Prospective policy analysis enables action-oriented policy research that is designed to inform future policy change, in this case, the strengthening of SSB taxes [25]. There have been a few comparative studies of SSB tax development, which have been retrospective policy analyses focussed on design and implementation [26,27]. This study builds on these analyses to make a contribution to methodological development with an additional focus on supporting systematic action-oriented research in low-resource settings. We first describe the study aim and objectives, as well as the theoretical frameworks that we drew upon to operationalize these objectives. We then describe the study method, including the process through which we made key decisions about study designs as a multi-country team.

## Study aim and objectives

The aim of the study was to analyse the policy landscape relevant to SSB taxation, in order to identify opportunities to strengthen SSB taxation-related policy for the prevention of nutrition-related NCDs in a subset of Eastern and Southern African countries: Botswana, Kenya, Namibia, Rwanda, Tanzania, Uganda, and Zambia. (Note the only exception to this was in Zambia, where SSB taxation is in place and therefore, the study was retrospective with respect to the first stage of implementation of SSB taxation [28]). This was achieved by establishing a comprehensive understanding of the political and evidence landscape in which policies for nutrition-related NCDs are being developed (or not developed), and to identify contextual factors (challenges and barriers) within the policy landscape that might influence design, adoption, implementation, and evaluation of SSB taxation and related policies.

The objectives of the study were:

1. To understand data availability on nutrition-related NCDs and associated dietary- and food-related indicators
2. To analyse the current policy landscape that would enable or challenge the prevention and control of nutrition-related NCDs (specifically, SSB-taxation and policies related to the use of revenue to increase access to healthy foods)
3. To describe relevant stakeholders and the political landscape, including the corporate political activity of SSB-relevant industries

### Rationale and theoretical frameworks underpinning study design

This study arose from the need for strong and effective policy to prevent the rising burden of NCDs in SSA countries. Robust health policies rely on evidence, but must also take into account the political, social, economic, and cultural context [29,30]. The selection of SSB taxation as the focal point for this study was based on recommendations by the World Health Organization (WHO) and other global public health bodies [31,32], particularly in light of the forceful opposition to the SSB tax implemented in South Africa in 2018.

We also considered the use of SSB tax revenue, particularly for supporting access to healthy food. Revenue from SSB taxes has been directed to a range of positive interventions in different contexts; such as French Polynesia (general prevention fund), Mexico (drinking water), and Philadelphia (preschool children’s education) [12]. In this project, we focused on ‘access to healthy food’ because accessibility to and provision of healthy food alternatives is a potential intervention to reduce consumption of unhealthy foods and beverages. Finally, we also explicitly considered activities of industry actors in the policy process, and their potential to assert political influence [33].

The implementation of an SSB tax in South Africa in 2018 highlighted the political and economic nature of NCD prevention policies, as well as the importance of strategic advocacy and evidence generation by public health actors [34,35]. It draws attention to the need to understand the data constraints, relevant politico-economic factors, key actors, and existing policy context relevant to SSB taxation in other countries. This study in Botswana, Kenya, Namibia, Rwanda, Tanzania, Uganda, and Zambia will thus contribute to (1) policy development regarding feasible, specific, and targeted evidence for the adoption of appropriate policies, and (2) the identification of specific recommendations for policy makers to strengthen policy for health that is tailored to the national context.

**Figure 1.**
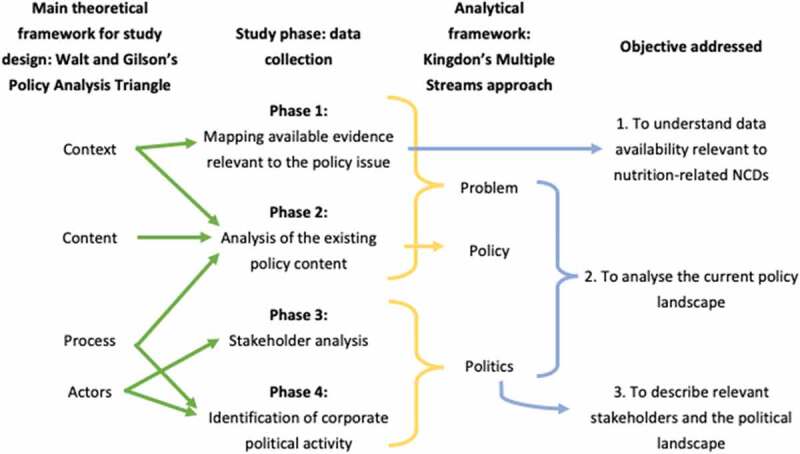
Illustration of the study design (frameworks, data collection, analysis) in relation to the study objectives

The design of this study was based on the Walt and Gilson health policy analysis triangle [30]. This framework was designed to inform health policy reform in low- and middle-income countries, and emphasises that in addition to the importance of evidence-based *content*, the political and institutional *context*, influential *actors*, and governance *processes* are important in shaping policy change. Figure 1 (left hand side) illustrates the way in which this framework informed the study data collection. The design of our pre-determined matrixes for systematic documentary data collection was also informed by theories from policy science and political economy that identify, in detail, the important influences on policy change [36–38]. These theories informed, for example, the inclusion of theory-derived columns to extract information on the framing of the policy problem and solution evident in the policy documents in our matrix for policy content. ‘Framing’ refers to the ways in which ideas regarding the nature of a policy issue and related actions are articulated, as well as the relative emphasis given to certain aspects of a given issue [39–41].

In designing the study tools for the documentary data collection and analysis, we also drew on resources from the WHO *Landscape analysis on countries’ readiness to accelerate action in nutrition*, which is designed to review challenges, opportunities, and possible actions to accelerate scaling-up of effective nutrition interventions [42]. This approach recommends collection of documentary (and other) data related to: political commitment, beliefs about nutrition, availability of evidence, the current policy, and legislative context and content, stakeholder interests and influence, and relevant food industry characteristics and activity.

In this study, we took an explicitly action-oriented focus in our analysis, as we sought to identify opportunities for policy change to strengthen policy for nutrition-related NCD prevention. Therefore, in order to underpin our data analysis, we drew on Kingdon's theory of agenda setting (the 'multiple streams' approach). Kingdon's theory focuses on factors that influence and can lead to specific instances of policy change, such as the strengthening of a SSB tax. Kingdon conceptualizes policy change as resulting from the interplay between three policy ‘streams’: the evidence for and perceptions of the policy problem (problem stream); existing policy and proposed solutions (policy stream); and political and institutional contexts (politics stream) [43]. The theory also highlights the role of influential policy actors in influencing policy change. Figure 1 (right hand side) illustrates the application of the analytical framework to the data collected in all four phases, and the relationship between the data collected, the analysis, and the achievement of the study objectives. Phase 1 directly achieves Objective 1, on data availability. The analysis drawing on Kingdon’s framework achieves Objective 2 (policy landscape) as well as Objective 3 (Stakeholders and political landscape).

In this study, we hypothesise that identification of opportunities to strengthen SSB taxation policy in SSA countries will be enabled by analysis of: existing policy content, the efforts of stakeholders to exercise power to shape policy in ways favourable to their interests, beliefs, and ideas, and available evidence regarding the nature of NCDs as a policy problem.

## Study methodology

### Overview

The study was conducted as a collaboration by researchers from nine institutions in South Africa, Kenya, Tanzania, Botswana, Rwanda, Namibia, Zambia, Uganda, and Australia. This unique collaboration was supported by a grant from the Government of Canada’s International Development Research Centre. The researchers met together in November 2018 for training in policy analysis research methods conducted by the first author, and to set the research focus and objectives.

The study design was based on the frameworks described above, using participatory techniques such as mind-mapping and electronic systems for voting on important inclusions in each phase of the study. To achieve the aims of the study in a systematic way across diverse low-resource settings, we developed a structured qualitative approach to documentary policy analysis, supplemented by consultations with key actors. Documentary policy analysis has previously been used in the health sector to examine both policy ‘actions’ and the implicit frames and actor influences evident in policy documents [37,44].

Data were collected in Kenya, Tanzania, Botswana, Rwanda, Namibia, Zambia, and Uganda between October 2018 and April 2019 via a desk-based review of relevant documentation, and verified through consultations with knowledgeable policy actors. Desk-based data collection was conducted systematically in four phases (Figure 1). To guide data collection for the four different aspects of the policy landscape the research team developed a set of matrix spreadsheets in Microsoft Excel ™. Data from each phase were first analysed separately; for example, the availability of data, and the policy content across sectors. We then analysed the data collectively in order to describe the policy landscape relevant to SSB taxation, using Kingdon’s Multiple Streams Approach to articulate the nature of the ‘problem’, ‘policy’ and ‘solution’ (Figure 1).

### Phase 1: mapping available evidence

Prior to the first project meeting, the research teams in each country mapped the availability of data sources to identify information in relation to nutrition related-NCDs. Subsequent to this, in the design of the protocol for Phase 1, the research team across all countries used mind-mapping and logic-modelling to identify a set of key variables from the relevant data sources, relevant to understanding the burden and context of nutrition related-NCDs. These variables addressed the following broad categories: food, diets, anthropometry, disease, health services (Table 1).

**Table 1.** Matrices and associated variables developed for the documentary analysis

Matrix 1: Data availability	Matrix 2: Policy content analysis	Matrix 3: Stakeholder analysis	Matrix 4: Corporate political activity (strategies)
Details of publication: Source, year of publication	Stated policy objectives	Nature of interest in the policy issue	Information and messaging
Diet: Fruit and vegetable consumption	Content relevant to nutrition-related NCDs (particularly SSB taxation and policies to increase access to healthy food)	Perceived level of interest	Financial incentives
Anthropometry: BMI, stunting, wasting	Framing: the problem and ‘appropriate’ solutions	Perceived level of influence/power	Constituency building
Diseases specific: Prevalence of NCDs and their risk factors	Governance: responsible sectors and coordination mechanisms	Source of power/influence	Legal
Health Services: NCD related morbidity and mortality, health care expenditure	Gender considerations (both in description of the problem and solutions)	Any evidence for the outcome/impact of their influence	Policy substitution
Economic: Food related taxes	Implementation		Opposition, fragmentation and destabilization
Demographics: Age, socio-economic status, gender			

These variables formed the basis of the column headings of Matrix 1 (Table 1) in Microsoft Excel ™. Data sources (rows) containing these relevant variables were then identified and entered into the matrix, and the relevant variables included in each data source were documented in the relevant column. The data sources were identified through a systematic search of websites and direct requests for information, focussed on the following:

- Government statistics office
- National Treasury
- University of Cape Town open data resource (DATA Project)
- WHO/United Nations agencies and other development partners
- Ministry reports (Health, Trade, Commerce, Agriculture, Education, Finance/budget statements, etc.)
- World Bank
- Industry data and Euromonitor
- Demographic Surveillance Site – Indepth Network
- Databases and other sources of obtaining literature presenting relevant work done in country (including searching local academic sources, as well as electronic databases such as PubMed, Embase, and Google Scholar)

### Phase 2: policy content analysis

The research teams in each country conducted a desk-based policy content analysis, to identify existing policy content and context. In this study, we focussed on public policy, which was defined as the means through which governments translate their ‘vision’ into action through various policy instruments. The policy sub-system of interest was defined as the prevention of nutrition-related NCDs, with a particular focus on SSB taxation and policies that increase access to healthy food. At the first project meeting, each team presented an overview of the existing policy and institutional frameworks relevant to nutrition-related NCD prevention in each country, based on searches of websites for policy documents and public statements. Data pertaining to legal frameworks and instruments were also collected and analysed [45].

The study team collaboratively developed a template matrix for policy content data collection and analysis (Matrix 2, Table 1). The matrix identified broad categories of ‘relevant’ policies in the rows, including: (1) *Whole-of-government* policy statements, such as the National Development Plan, to identify the level of priority given to NCDs and the roles of various line ministries (sectors) in policy and action on NCDs; (2) *Sectoral* policy documents (i.e. Health, Agriculture, etc.), such as the national nutrition policy, in which we examined commitments specific to SSB taxation and policies to increase access to healthy food; and (3) *Implementation*-focussed documents, which guide action, such as the administering documents relating to excise taxation. The column headings in the matrix included policy objectives and content relevant to nutrition-related NCDs and additional contextual factors. Based on our study frameworks (see above), the contextual factors included the ways in which the policy problem(s) and solution(s) were framed in the policy documents, the governance and resourcing arrangements, and responsibilities for action. Using this pre-determined matrix as a reference enabled the study teams to identify the existing policy content, as well as the gaps in policy content in a systematic way.

We identified relevant policy documents initially through searches of Government websites and other relevant institutions, as well as legal institute websites operating in each country, using search terms based on the relevant sectors the study team identified (e.g. health policy, agriculture policy, nutrition policy). We then used the snowballing method, based on mentions of relevant guidelines, strategies, policies, and action plans in other policies, and also made direct requests to relevant Ministries.

### Phase 3: stakeholder analysis

We conducted a desk-based stakeholder analysis to identify key actors with potential influence on the adoption of SSB taxes. For this study, we defined stakeholders as: *‘actors who have an interest in the issue under consideration, who are affected by the issue or who – because of their position – have or could have an active or passive influence on the decision making and implementation processes. They can include individuals, organizations, different individuals within an organization, and networks of individuals and/or organizations, i.e. alliance groups.’* [46:p341]. Relevant stakeholders in this case included actors and institutions from government, civil society, soft drinks/beverages industry, and academia.

In designing the protocol for Phase 3, the research team mind-mapped different types of potential stakeholder groups, as well as mapping potential stakeholders based on the issue. We developed Matrix 3 (see Table 1) for stakeholder analysis, following Varvasovzsky and Brugha’s approach [46], to document the attributes of identified stakeholders, their interests and influence. We identified relevant information through searching websites of relevant government sectors, organizations, and institutions operating in the country, as well as searches of academic literature, using the terms from the matrix and the types of relevant stakeholder groups as search terms (e.g. health NGO, SSB companies). We also drew on the policy content analysis in phase 2, to identify stakeholders engaged in policy development and implementation. Each identified stakeholder formed a row in the matrix, and information collected was entered into the relevant columns (Matrix 3, Table 1).

### Phase 4: industry corporate political activity

We conducted an analysis of corporate political activity by the SSB-related industry, adapting the method developed by Mialon and colleagues [33]. Corporate political activity was defined in this study as actions undertaken by the SSB industry that have been identified as *‘corporate attempts to shape government policy in ways favourable to the firm’* [33]. Matrix 4 (Table 1) was developed based on this framework to guide data collection, with each industry actor entered as a new row, and relevant information and evidence regarding their corporate political activities entered into the relevant columns.

Each countries’ research team drew on the stakeholder analysis (Phase 3) to identify the key industry players in each country. We collected data relevant to each study country through a comprehensive online search for existing and future trends in SSB sales and consumption, market dynamics, and industry tactics. Data sources included food industry websites and reports, government reports, media articles, and industry analytical reports including (purchased) Fitch Solutions industry reports for each country (with the exception of Rwanda).

### Consultations

To complement the desk research in each phase, each study team conducted consultations in order to (1) verify that we had identified all relevant policy documents; (2) ensure that we had correctly interpreted the policy content. Consultations were voluntary, and were strictly focused on publicly available information that was part of the participants’ job description, normal work, and responsibility. Relevant stakeholders were identified through the desktop review. In three study countries (Uganda, Rwanda, and Tanzania) the first author conducted consultations with several key individuals involved in relevant policy development and implementation. In four countries (Zambia, Namibia, Botswana, and Kenya) the consultations were conducted as part of in-depth semi-structured key informant interviews with key stakeholders. The methods for the additional qualitative research in these countries (including tools, sampling, ethical approval, and analysis of interviews) are described in detail within individual country-specific papers [28,47,48].

### Data analysis

We qualitatively analysed the policy landscape relevant to SSB taxation, drawing on our study frameworks, in order to identify opportunities to strengthen SSB taxation-related policy for the prevention of nutrition-related NCDs. The first step of data analysis focussed on each individual matrix, identifying strengths and opportunities (gaps) in evidence availability, framing, and beliefs evident in policy content, stakeholder interests, and political activity by industry actors. This analysis was conducted using the rows and columns of each matrix, to assess the strengths and gaps; for example, analysis by rows identifies strengths or gaps within data sources or policies, or for different stakeholders, and analysis by columns provides an analysis across all sources for each component (e.g. framing of the policy problem, desired data, industry tactic, etc.).

The second stage of analysis was undertaken using Kingdon’s Multiple Streams approach, drawing on the data collectively (Figure 1). Within the ‘problem’ stream, we analysed the documentary data, complemented with information provided through the consultations, with a focus on issue framing and perceptions of the problem, existing evidence on the problem, and the extent to which sugar, SSBs and nutrition-related NCDs are regarded as a problem. Under the ‘policy’ stream, we analysed the extent to which existing policies address the challenge of nutrition-related NCDs, the governance mechanisms, sectoral interests and priorities, and frames regarding policy solutions for NCDs. In the ‘politics’ stream, we analysed actor power and influence, industry tactics, and government priorities. The results section of each of the country papers are a write up of this second stage of analysis [28,47–50].

### Gender, study design and analysis

The study team was committed to considering gender at all stages of the research. Presently women, especially urban women, are more likely to be overweight or obese, and are disproportionately affected by NCDs in SSA [51,52]. In documenting evidence availability, the availability of gender-disaggregated data for relevant variables and indicators was observed and noted. Similarly, for each policy document reviewed, we noted any mention of gender in the articulation of the policy problem and solution.

## Reflections on the study design and methods

This prospective policy analysis study design enabled us to generate a comprehensive overview of the policy context, policy content, and the political economy relevant to SSB taxation in seven SSA countries. The mapping of evidence availability was an extremely useful addition to traditional policy analysis because of the importance of evaluation and evidence in health policy [25,37].

Based on the data collected, six of the seven country research teams were able to identify specific opportunities to strengthen SSB taxation-related policy for the prevention of nutrition-related NCDs. These country case studies [28,47–50] addressed the specifics of policy content. They also identified specific opportunities to strengthen SSB taxation for NCD prevention, such as increasing the tax rate currently applied to SSBs in Uganda [50], or amending the target of taxation to exclude non-SSBs such as bottled water in order to maximise the health impacts of the tax in Rwanda [49]. In addition, the study design also enabled the research team to analyse the data across countries to identify learnings at a regional level, taking into account the political and politico-economic context, particularly as it relates to regional trade and industrialisation policies in SSA [48,49,53]. The research design also supported legal analysis across countries of the regulatory documents identified [45].

## Study strengths and limitations

In practice, obtaining policy documents was straightforward; the approach of using internet sources together with direct approaches to relevant ministries was successful. Budget speeches were also a useful source of data, in terms of identifying the explicit rationale for taxation. The main limitation of this desk-based policy analysis study design is that only limited information on frames and paradigms related to the policy problem and the policy solution (SSB taxation) can be inferred from policy documents, even though they are hugely influential in policy making.

In addition, the data provided only indicative evidence on the outcome of stakeholder influence and there was also limited documentary evidence for corporate political activity, which reflected findings in other low and middle-income countries [54]. In Namibia, for example, industry documents were not available online and our direct approaches to industry either received no response or were refused [48]. In contrast, in Uganda there was useful information on political activity from the online articles by major media outlets [50]. The addition of qualitative policy interviews in three countries was very helpful in triangulating documentary information and enabling further exploration of power and influence, as per case study research methodology [55].

The consultations and key-informant interviews were useful in: confirming that all relevant documents had been identified; providing the researchers with contextual background to the policy process; and in identifying potential forthcoming policy considerations, which were then confirmed by drawing on other documentation (such as budget speeches). They were also useful in categorising the different stakeholder interests in the stakeholder power/interest matrix.

## Conclusion

This study design paper reports on the development and operationalization of a policy landscape analysis, in the form of a prospective documentary policy analysis focussed on strengthening SSB taxation in Uganda, Rwanda, Zambia, Namibia, Botswana and Kenya, and Tanzania. The study design built on previous analyses to develop an approach to action-oriented policy analysis feasible in low-resource settings. Analysing availability of data, policy content, stakeholders and corporate political activity enabled the study team to identify opportunities to strengthen SSB taxation in each country, and to understand the politico-economic context for SSB taxation in the region. Research that examines the policy content and political context can provide insights to inform strategic public health advocacy action to support SSB taxation in the region. By enabling comparable research across the region, this study design could also support the development of a policy roadmap for the region that is rooted in a nuanced understanding of existing government priorities and actions a well as the interests of influential stakeholders.

## Paper context

Sugar-sweetened beverage taxes are recommended as part of comprehensive action to prevent non-communicable diseases, but so far there has been limited uptake in sub-Saharan Africa, despite rising non-communicable disease prevalence in the region. This paper describes the collaborative development of a method for prospective policy analysis, a ‘landscape analysis’, conducted in seven sub-Saharan African countries in 2018–19. The analysis is based on systematic documentary analysis supplemented by consultations, and is feasible in low-resource settings.
